# Tracing Microplastics in the Human Body: From Detection to Disease Mechanisms

**DOI:** 10.3390/diagnostics15232971

**Published:** 2025-11-23

**Authors:** Stefana Anastasia Talau, Mihaela Chialda, Cristian Ichim, Horatiu Dura, Ciprian Tanasescu

**Affiliations:** Faculty of Medicine, “Lucian Blaga” University of Sibiu, 550024 Sibiu, Romania; stefanaanastasia.talau@ulbsibiu.ro (S.A.T.); horatiu.dura@ulbsibiu.ro (H.D.); ciprian.tanasescu@ulbsibiu.ro (C.T.)

**Keywords:** microplastic, inflammation, cancer, respiratory tract

## Abstract

Microplastics (MPs), defined as plastic particles < 5 mm diameter, have become a growing public health concern. First identified in the aquatic environment in 2004 and later in air samples in 2015, airborne MPs display wide variations in shape and size, with fibres being the most common. These physical characteristics, together with others such as median aerodynamic diameter, influence how deeply they penetrate and where they deposit within the respiratory tract. Recent studies have confirmed the presence of MPs in nasal lavage fluid, bronchoalveolar lavage fluid, sputum, pleural fluid and lung tissue samples, with higher concentrations observed in older individuals, smokers and those with occupational exposure. Multiple polymer types have been identified, most frequently polypropylene, polyethylene and polyester. Experimental models demonstrate that MPs can induce inflammation, oxidative stress, mitochondrial dysfunction, microbiota alterations, fibrosis and carcinogenic changes, with toxicity generally increasing as particle size decreases. Despite the growing evidence of plastic toxicity, only a limited number of studies have examined MPs’ influence on the respiratory system, focusing mostly on polyester spheres, rather than fibres, which dominate real-world exposure. Current findings suggest MPs contribute to several pathophysiological processes and may play a role in respiratory disease. However, further research is needed to clarify the underlying mechanisms, long-term consequences and clinical relevance of these emerging pollutants.

## 1. Introduction

Plastic materials first appeared in the 1950s, and since then, their production has increased steadily. Their usage has become deeply embedded in daily life, largely driven by industrialization and urbanization in recent years [1,2,3]. Plastic production exceeded 400 million tons in 2023, representing increases of 105% compared to 2000, 51.85% compared to 2010 and 9.33%. compared to 2020. Recent efforts toward recycling and reuse have begun to slow production growth, with the United Nations Environment Programme (UNEP) aiming for an 80% reduction in plastic output by 2040 [4]. The plastic production rate has slowed down while scientific concern about its effects on the environment and health has risen.

Microplastics (MPs), described as plastic particles with a diameter less than 5 mm, produced as such or resulting from the degradation of macroplastic, were first described in 2004 by Richard Thompson, a marine ecologist [5,6,7]. More recently, mesoplastics (MesPs) have been classified as plastic measuring from 1 to 10 mm, MPs as 1–1000 μm and nanoplastics (NPs) as particles < 1 μm [8,9,10]. However, these dimensional thresholds are not universally standardized, and definitions can vary slightly between environmental and toxicological studies. Microfibres (MFs), a specific subset of MPs characterized by their elongated, thread-like shape, are commonly derived from synthetic textiles and represent one of the most abundant forms of airborne MPs [11].

Plastics encompass a diverse group of synthetic polymers with distinct chemical structures, physical properties and environmental behavior. Polymers such as polyethylene (PE), polypropylene (PP), polystyrene (PS), polyvinyl chloride (PVC), and polyethylene terephthalate (PET) dominate global production and differ in monomer composition, crystallinity, flexibility and thermal stability [12]. These intrinsic variations influence how plastics fragment into MPs/NPs and determine their physical properties. Additives, including plasticizers, stabilizers and dyes, further contribute to heterogeneity by leaching during degradation. As polymer chemistry governs both particle formation and biological reactivity, outlining these distinctions is essential for understanding their environmental distribution and health implications.

Microplastic pollution is widespread in the atmosphere, hydrosphere, biosphere and lithosphere, posing increasing risks to ecosystems, biodiversity and human health, although many effects remain insufficiently characterized [12,13]. This burden is driven by decades of high plastic production, inadequate waste management, suboptimal treatment processes and the environmental persistence of plastics, which degrade slowly into micro- and nanoscale debris [7,8,12,13,14]. Humans are exposed to MPs/NPs through ingestion, being found in many types of cooking products, through inhalation and dermal exposure, but once internalized, these particles may translocate to distant tissues via the circulatory system [12,15,16,17]. The estimated weekly human exposure to MPs ranges from 0.1 to 5 g [18].

Most commonly, MPs act as carriers for environmental contaminants, including heavy metals, polycyclic aromatic hydrocarbons and antibiotics [10,18,19,20]. A study that analyzed the MPs from Pearl River identified multiple heavy metals within the MPs, such as arsenic (As), zinc (Zn), nickel (Ni), cadmium (Cd), iron (Fe), copper (Cu) and manganese (Mn), with Fe, Zn and Mn having the highest concentrations [7]. MPs’ ability to adsorb environmental pollutants is enhanced by surface abrasion, broken edges, crushing or a squelched appearance, some of these modifications also occur due to photodegradation which changes the molecular structure, making it more prone to faster degradation [7,21].

Humans are primarily exposed to MPs and NPs through ingestion and inhalation, while dermal contact has been suggested as a possible, but less substantiated route. Evidence indicates that sweat composition may influence nanoplastic aggregation and skin penetration through follicles and glands [22]. Broader reviews describe dermal uptake as a potential route, but largely unquantified, compared with the well-documented respiratory and digestive exposure pathways [23]. As this review focuses on airborne and respiratory exposure, the dermal route is only briefly mentioned here for completeness.

Considering the importance of plastic usage in our daily life, their effects remain insufficiently studied, and there is high interest in the research field. We aim with this review to summarize the knowledge about airborne microplastics and to emphasize the gaps in the literature.

Studies were included if they investigated the presence, characteristics, or biological effects of MPs in the air, human respiratory system or in experimental animal models relevant to human respiratory exposure. Eligible human studies analyzed MPs in nasal lavage fluid (NLF), bronchoalveolar lavage fluid (BALF), sputum, pleural fluid, or lung tissue, using validated analytical techniques. Experimental studies on animals were included only when they addressed pathophysiological mechanisms.

Exclusion criteria included studies lacking explicit identification or quantification methods. Non-English publications and studies with unclear contamination control procedures were also excluded.

## 2. Atmospheric Exposure to Microplastic—Inhalation

MPs and NPs are present in the atmosphere across a wide range of environments. Their concentrations are highest in densely populated urban areas but are also detectable in suburban regions with lower human activity. In contrast, rural areas generally show substantially lower atmospheric MPs/NPs levels [24,25,26,27]. Air quality tends to be poorer in urban areas, meaning a higher Air Quality Index; one reason for this may be the concentration of MPs/NPs in the atmosphere, even though quantifying this contribution remains challenging [28,29]. A study conducted in Central London found 20 times higher values of airborne MPs than in French Pyrenees, indicating that population density is correlated with the amount of released MPs [30].

Indoor environments often contain even higher MP concentrations than outdoor air. Dris et al. in 2016 showed that 355 plastic fibres/m^2^/d were released into the atmosphere, and in 2017, they discovered MP particles in indoor environments, reporting concentrations ranging from 0.4 to 59.4 fibers/m^3^, suggesting that indoor MPs may represent a major source of exposure to airborne microplastic [31,32]. Moreover, indoor MPs also tend to exhibit larger particle sizes, making them less likely to be inhaled deeply into the lungs [31,32]. The most common microplastic types in indoor air are polyester (PES), followed by PE [26,33]. Microplastic can be released indoors by carpets, clothing, furniture and building materials, among others [15,34,35].

Several factors influence indoor MP deposition. Momeni et al. demonstrated that residence type, building characteristics and occupational exposure (house cleaners, laundry workers, tailors, textile sellers and weavers) affect the deposition of MPs based on shape [27]. Textiles represent one of the major contributors to environmental MPs, accounting for approximately 34.8% of total MPs release, predominantly in the form of MFs [7,11]. Occupational exposure has been documented in industrial settings, where Wright et al. reported high airborne concentrations of polyamide (PA) and PVC microplastics (0.8/mL and 0.5/mL, respectively) [26,36].

Besides building type, the floor level also influences exposure. Ageel et al. showed that participants living on upper floors had significantly higher MP concentrations in bronchoalveolar lavage fluid (BALF) (4.48 ± 0.31 items/100 mL) compared to those residing on the ground floor (3.44 ± 0.17 items/100 mL), suggesting that human activity and indoor air circulation can resuspend and elevate MPs from lower surfaces [37,38].

### 2.1. Microplastic Release and Human Exposure from Face Masks

The COVID-19 pandemic has led to the widespread use of disposable and reusable face masks, which, while essential for infection control, have emerged as a potential source of airborne MP, NP and MF exposure. This dual role, protective against pathogens, but contributory to synthetic particle inhalation, has generated increasing scientific and public concern, especially among healthcare workers and other professionals who use masks for extended periods.

Studies have shown that wearing either cotton or surgical masks decreases the inhalation of spherical MPs and increases the inhalation of fibrous MPs, possibly due to the trapping of spherical MPs onto the pores of the masks [39]. The main polymers found in these masks are PP, PA, PE, polyurethane (PU), PS, PET and PES, with PP being the predominant component [40,41,42]. Studies have shown that surgical masks release more MPs compared to other types, especially if they are improperly stored, are used for a prolonged period of time, and the material is aged; under such conditions, masks can release up to 1 billion MPs [26,43,44,45,46,47]. Moreover, a 90% decrease in exhaled particles has been observed in mask wearers, which may contribute to MPs accumulation in the respiratory tract over time [48,49]. Although these findings raise legitimate occupational health questions, masks remain indispensable for controlling airborne pathogens. Their role as secondary MPs sources underscores the need for improved design and material standards. Reusable masks made from natural fibres may reduce MPs release, but they require proper laundering and replacement protocols to prevent microbial contamination.

In summary, face masks represent a complex intersection between infection prevention and microplastic exposure. Future studies should explore alternative materials that balance respiratory protection with reduced particle emission.

### 2.2. Microplastics Physical Features

Airborne microplastics display substantial variability in shape, size, and aerodynamics, largely influenced by regional sources and sampling conditions. Several studies have shown that particles measuring 10 μm or less can be inhaled and deposited in the human respiratory tract [15,40,50]. For example, in London, UK, fragments represented 64% of all detected airborne MPs. However, it is known that the predominant types of MPs in biological samples are fibres and fragments [17,36].

The shape of a microplastic strongly influences its deposition behavior. Elongated particles have a higher velocity movement when their long axis aligns with the direction of airflow, increasing the likelihood of reaching deeper regions of the respiratory tract, while spherical particles can distribute more evenly and deeply due to their small size [27]. On the other hand, particles with a mass median aerodynamic diameter (MMAD) less than 2.5 μm are considered respirable, and those with an MMAD ranging from 2.5 to 100 μm are considered inhalable, so the first category can penetrate deeper into the lung tissue due to the lower MMAD [20].

Across global studies, MP size distributions vary widely. In Paris, France, MPs ranged from 100–5000 μm range; in Tehran, Iran the microplastic particles measured mostly between 250 and 500 μm; and in Dongguan, China, the majority of MP/NP particles had dimensions between 100 and 500 μm, while the maximum size exceeded 3500 μm [51,52,53,54]. Additionally, air samples collected from public transportation, residential areas and workplaces contained MP particles smaller than 100 μm, posing an inhalation risk to human beings [15,55]. Importantly, human tissue studies reveal substantially smaller particles: Luis Fernando et al. reported polymer particles < 5.5 μm and fibres measuring 8.1–16.8 μm in lung samples [13]. Furthermore, in California, a study determined that the most predominant length category of MPs was 101–301 μm, regardless of whether the sample was from indoor or outdoor air, while particles with a diameter below 130 μm can potentially translocate and accumulate in human tissues [27,56].

Microplastics exhibit diverse morphologies, most frequently categorized as fibres or fragments. Fibres are elongated, thread-like microplastics, typically originating from textiles or synthetic fabrics, while fragments display irregular, angular shapes derived from the breakdown of larger plastic materials [11]. In this review, the term “particle” is used in a general sense to refer to microplastic items for which shape was not specified in the original studies, and therefore does not necessarily imply a spherical morphology, when shape was reported, it is explicitly stated. This clarification is important to avoid confusion, as many studies report total microplastic counts without differentiating shapes, even though aerodynamic and deposition properties differ substantially between fibres and non-fibrous fragments.

Color is another identifiable physical feature. Jahedi et al. found that MPs recovered from the human respiratory tract were predominantly transparent or white [17,27]. Table 1 summarizes primary studies on airborne microplastics, highlighting the dominance of fibres among the detected shapes, along with the diversity of sizes and polymer types identified in air samples.

### 2.3. Nasal Lavage Fluid

The first anatomical filter of the inhaled air is the nasal cavity, trapping airborne particles of at least 5 μm, blocking larger particles, while smaller particles may bypass this barrier and reach deeper regions of the respiratory tract, as reflected by lung tissue findings reporting particles between 1.60 and 16.80 μm [13,48,57].

Studies analyzing nasal lavage fluid (NLF) confirmed upper-airway exposure to MPs with abundances of 1–2.38 particles/mL [58]. Jiang et al. studied microplastic exposure for both indoor and outdoor workers, and they found more MPs in the NLF of indoor workers (87.0%) compared to the outdoor workers (83.8%); the particles were mostly fibrous, and the predominant types of MPs for outdoor workers were PA (25.3%) and PE (22.9%), while for the indoor workers, they were PVC (41.1%) and PA (31.6%) [59]. The difference between the most common microplastic types might be explained by the difference of exposure in the work environment: the indoor workers are mostly surrounded by furniture, containing usually PVC, whereas the outdoor workers used masks during COVID-19 pandemic, consisting mostly of PE [59].

Multiple studies investigating mask wearers observed substantially elevated NLF microplastic levels compared to non-wearers, reporting 14–67.3 particles per 5 mL—approximately five-fold higher—along with smaller particle sizes (<100 μm) and a predominance of fibres [43,48,60]. These findings suggest that NLF effectively reflects recent or short-term exposure to airborne MPs, serving as an early indicator of inhalation.

### 2.4. Broncho-Alveolar Lavage Fluid and Sputum

BALF and sputum analyses provide insight into the clearance and retention of inhaled MPs within the lower respiratory tract. Most studies that analyzed sputum reported an abundance of between 468–156 particles/100 mL and found that over 90% of MPs are fibrous and measure mostly <100 μm, but also some extreme dimensions were found, for example, 10.5 mm [17,27,40,59,61]. Dominant polymers include PC, PVC, PA, PU, and PES, typically appearing white or transparent, consistent with indoor air sources [17,27,40,59].

Factors such as smoking, occupational exposure, mask-wearing frequency, and recent invasive procedures were associated with higher MP counts and more diverse polymer types [17,27]. Huang et al. found that patients with recent invasive tracheal examination had a higher amount of alkyd varnish in their sputum sample, suggesting that the coating of the fibreoptic bronchoscope may leave residue behind [40].

BALF studies reported more rare MPs, similar shapes, but slightly larger particle sizes, with fibres > 100 μm and a higher proportion of fragments and spheres, the longest measuring 1.135 mm [17,27,62,63]. The predominant polymers detected in BALF, PE, PET, PA, PU, and rayon/viscose, indicate contributions from both synthetic and semi-synthetic materials [17,20,27,37,62,63].

Altogether, BALF and sputum investigations demonstrate that inhaled MPs can deposit and persist within the lower tract, but the detection of such particles in lung tissue further suggests long-term accumulation beyond mucociliary clearance.

### 2.5. Pleural Fluid and Lung Tissue Samples

Investigations of lung tissue and pleural fluid provide compelling evidence for chronic MP retention and cumulative exposure. The earliest report, published by Pauly et al. in 1998, documented synthetic fibres embedded in human lung tissue [64]. Subsequent analyses, using μRaman and μFTIR spectroscopy, detected MPs in 65% of samples, mostly fragments (1.6–5.56 μm) and fibres (8.12–16.80 μm), predominantly PP and PE [13].

More recent work identified PP, PET and PE as dominant types, more commonly fibres, measuring up to 2475 μm in length, particularly abundant in lower regions [65]. Comparative analyses of tumoral vs. non-tumoral tissue revealed that tumoral samples contained approximately twice as many MPs, with longer particles (mean length 1.45 ± 0.98 mm) and higher proportions of PET and rayon [66]. The abundance of MP fibres was higher in older patients, supporting the idea that MPs accumulate gradually over time, with increasing age [37,66].

Pleural fluid investigations report polymer profiles similar to those found in lung tissue, with PES and PA being the most frequently detected types, although fragments and spheres appear proportionally more common than in BALF or sputum [27].

Table 2 summarizes key studies that have detected microplastics in various human respiratory samples, including NLF, sputum, BALF, pleural fluid, and lung tissue. For each study, the analytical methods, sample type and main polymer types of detected microplastics are presented. These findings collectively demonstrate that inhalation is a significant route of microplastic exposure, with particles being detected from the upper airways to the deep lung regions. The diversity of analytical techniques and sampling approaches also highlights the current methodological heterogeneity and the need for standardized detection protocols in future research.

Although microplastics have been consistently detected in respiratory samples such as NLF, sputum, BALF, pleural effusions and lung tissues, these findings must be interpreted with caution. Reported abundances and polymer profiles are strongly influenced by the analytical sensitivity, sampling procedures, and contamination-control measures used in each study. Variations in digestion methods, filtration materials, and polymer identification techniques can lead to substantial differences in detection rates and particle profiles. Thus, while these observations provide strong evidence of airborne exposure and tissue deposition, their quantitative accuracy remains limited. The methodological constraints underlying these measurements are further detailed in Section 3.

## 3. Methodological Constraints in MPs Detection

Despite increasing evidence of MP presence in respiratory samples, significant methodological constraints limit both the comparability and validity of published findings. These challenges arise from variations in sampling conditions, digestion and filtration protocols, polymer identification methods and contamination control practices, all of which may influence the reported abundance and composition of microplastics. For this reason, a rigorous assessment of methodological quality is essential. Five critical domains determine analytical accuracy: contamination control, blank validation, spectroscopic identification, tissue integrity and analytical bias.

### 3.1. Cross-Contamination and Clean Lab Procedure

Cross-contamination remains one of the most critical methodological issues in microplastic analysis. Airborne fibres, laboratory clothing, and laboratory plastics can easily contaminate samples during collection or processing. Nevertheless, only a minority of studies (38%) report working under controlled air conditions. Experiments conducted in laminar flow hoods have been shown to reduce contamination by up to 97% compared with open laboratory environments, whereas fume hoods are largely ineffective, as they draw unfiltered air from the room. Ideally, all procedures should be carried out in laminar flow hoods or clean rooms with HEPA filtration and restricted access [67].

Additional preventive strategies include the use of cotton lab coats, the exclusive use of glass or metal equipment and pre-filtering all reagents through membranes with pore sizes equal to or smaller than those used for sampling. Thermal treatment of glass fibre filters (450 °C for ≥3 h) effectively removes pre-existing fibres and should be systematically adopted [67].

### 3.2. Blank Controls and Correction Strategies

Alongside minimizing airborne and procedural contamination, the systematic use of blank controls is essential for assessing the true extent of cross-contamination and ensuring data reliability. Procedural blanks, air-deposition controls, and field blanks are essential for identifying and correcting contamination in microplastic analyses [67]. However, their implementation remains inconsistent: only 60% of studies include procedural blanks, 34% incorporate air-deposition controls and just 12% employ both [67].

Reported contamination varies widely—from 0 to 36 particles per blank (mean 4)—with the greatest burden in smaller size fractions (22 MPs of 20–50 μm, 11 of 50–100 μm, 3 of 100–500 μm, and <1 of 500–1000 μm) [67]. Even under laminar-flow conditions, contamination persists, for example 27 fibres were detected in procedural blanks and 19 fibres in open Petri dishes over three weeks [68]. In another study, field blanks, filters handled and mounted like real samples, contained 323 fibres and 319 particles (*n* = 3), values comparable to indoor and outdoor samples, confirming pre-sampling contamination [68]. Additional sources included sampler inlets and filter handling, introducing ~106 fibres—far exceeding contamination acquired during laboratory processing alone (27 fibres) [68].

Typical contamination sources include cotton lab coats, paper towels, and cross-sample transfer [67,68]. For reliable quantification, procedural, field, and air-deposition blanks must run in parallel with real samples, and contamination values should be transparently reported and, where justified, subtracted. Standardized blank correction approaches are critical to enable valid inter-study comparison.

### 3.3. Spectroscopic Identification Limits

Even when contamination is minimized, accurate identification of microplastics remains challenging due to analytical constraints. Most studies use μ-FTIR or Raman microscopy, sometimes complemented by SEM-EDS. These techniques differ in detection limits and sensitivity: μ-FTIR typically detects particles ≥ 10 µm, whereas Raman microscopy can reach <1 µm, but is often affected by fluorescence from pigments and additives [69].

Methodological choices significantly influence reported microplastic abundance. According to Kwon et al., methodological choices greatly influence reported abundances. Fully automated μ-FTIR workflows have overestimated particle counts up to fivefold because of false positives, while manual approaches missed small or transparent particles. The semi-automated method, which combine ultrafast mapping with algorithmic classification and manual verification, offers the best compromise between accuracy and efficiency. Raman microscopy excels at detecting small particles (<50 μm) but is slower and more vulnerable to spectral interference, while μ-FTIR provides more stable spectra for larger or irregular particles [69].

These methods are complementary, not interchangeable. Standardized spectral libraries, harmonized acquisition parameters, and validation of automated algorithms are essential to ensure comparability and data reliability [69].

### 3.4. Validity of Post-Mortem Samples

While spectroscopic techniques such as μ-FTIR or Raman microscopy have greatly improved the precision of microplastic identification, the accuracy of these analyses ultimately depends on the quality and integrity of the biological material. Post-mortem human tissues offer a rare opportunity to directly evaluate respiratory deposition, yet they present substantial methodological vulnerabilities.

Contamination may occur during dissection, fixation, paraffin embedding, or storage, potentially introducing exogenous particles or altering the chemical signature of endogenous ones. Furthermore, determining whether detected microplastics reflect ante-mortem exposure or post-mortem contamination is challenging. Two μ-FTIR-based studies reported microplastics in 11 of 13 lung samples and microfibres in 100 surgical specimens, both emphasizing contamination risk and size detection limits [65,66]. Thus, rigorous sampling documentation and clean dissection environments are essential to strengthen confidence in post-mortem data and minimize biases.

### 3.5. Limitations and Biases

Despite advances in analytical precision, significant methodological limitations persist in microplastic research, particularly concerning contamination control and data correction. Even under controlled laboratory conditions, cross-contamination can artificially elevate particle counts. While procedural and field blanks help monitor this contamination, blanks themselves carry uncertainty. Differences in sampler surface area, exposure duration, and discrepancies between blank matrices (often ultrapure water) and complex biological tissues can introduce systematic errors during blank correction. Inconsistent correction approaches contribute to variability and may result in either over- or underestimation of microplastic abundance [70].

Analytical constraints also add to quantification bias. ATR-FTIR has limited sensitivity for small particles, Raman spectroscopy suffers from fluorescence interference, and Nile Red staining may generate false positives from non-plastic organic matter. Moreover, discrepancies in recovery efficiency across matrices and extraction chemistries highlight the difficulty of standardizing protocols [70].

To minimize these biases, it is crucial that future studies explicitly report blank characteristics and results, standardize corrections by sampler area and exposure time, define and validate detection limits, and provide full transparency in quality assurance procedures. Such harmonization would strengthen data comparability and improve the reliability of conclusions regarding human exposure and health risk [70].

Methodological inconsistency is compounded by the absence of unified regulatory standards. Casella et al. highlight that current legislation is fragmented, with uneven definitions, metrics, and monitoring requirements for MPs across regions, impeding inter-study comparability and risk assessment. Convergence of analytical quality assurance and quality control with regulatory harmonization is therefore essential [71].

## 4. Health Effects of Microplastics on the Respiratory System

The human respiratory system is continuously exposed to airborne MPs and NPs, with estimates suggesting up to 3.0 × 10^7^ particles inhaled annually. Although most are cleared by mucociliary transport, smaller particles are able to bypass this defense and accumulate in lung tissue [28,58,72,73]. A recent histological study detected an average of 1.42 ± 1.50 MPs/g of human lung tissue [65]. Particles < 5 μm can penetrate into distal airways, while larger ones are retained in the upper airways, for example, a 135 μm particle is about a quarter of the diameter of a 17-generation bronchus [12,20,26]. Ultrafine particles (<2.5 μm or <0.1 μm) may reach the alveoli, where their clearance becomes limited [74]. Width rather than length determines inhalability, fibres between 13–125 μm in diameter have been detected in lung tissue, whereas long, thin particles are more likely to persist [66,74]. Polarity also influences deposition: polar particles favor upper-airway retention, whereas non-polar ones migrate deeper into the lower airways [75].

Microplastics also act as “Trojan horses” by adsorbing toxic additives and environmental pollutants such as phthalates, bisphenols, and heavy metals, amplifying their toxicological impact and promoting cellular damage and inflammation [7,10,17,20,59,76,77,78,79]. Because additives are not bound to the matrix of the polymer, they can migrate to the surface of the particles and potentially traverse the alveolar-capillary membrane and enter the systemic circulation [80]. Phthalates interfere with alveolar maturation and gene expression, while bisphenols alter alveolar morphology and induce allergic responses, including asthma [80]. Chronic occupational exposure to PP, PVC, PA and PES has been associated with cough, dyspnea, wheezing and increased lung cancer risk [28,40,48]. Nylon and PVC exposure has also been linked to interstitial lung disease [13,20]. Increasing evidence suggests that MPs may contribute to asthma, pneumoconiosis, and other chronic airway disorders [81,82,83].

The toxicological impact of microplastics is influenced by their physicochemical characteristics. Smaller particles, owing to their greater hydrophobicity and deeper penetration into distal airways, tend to induce stronger cytotoxic and inflammatory responses [12,13,20,65,84]. Even extremely small particles (~50 nm) can produce cytotoxic and genotoxic effects in macrophages and lung epithelial cells [85,86]. Shamsul Anuar et al. demonstrated that MP exposure alters tracheal contractility, producing an exaggerated response to muscarinic stimulation [87]. This dysregulation of airway tone may impair airflow control and, in patients with asthma or COPD, can contribute to mucus hypersecretion and bronchoconstriction, exacerbating disease severity [87]. Further studies reported that MP exposure disrupts the barrier integrity of the respiratory epithelium and impairs the development of human airway organoids, increasing susceptibility to irritants, allergens, and microorganisms, and thereby predisposing to rhinitis, chronic rhinosinusitis, and asthma [79,88].

At tissue level, MP exposure promotes macrophage aggregation, altered metabolism and adhesion, and protein expression changes mediated by p38 signaling and Toll-like receptor 2, contributing to apoptosis and inflammation [13,26,73,74,89]. Macrophages can internalize MPs via endo- and exocytosis, but phagocytosed MPs are not degraded, instead forming granulomas and maintaining a chronic inflammatory state [20,75,85].

Tomonaga et al. further showed that mice exposed to PP MPs developed dose-dependent lung inflammation, with increased total cell and neutrophil counts, elevated myeloperoxidase and LDH activity, and upregulated chemokine genes such as CD177 (involved in neutrophil migration and activation), LCN2 (involved in neutrophil recruitment), CXCL1 (activate neutrophils and act as chemoattractants) and CXCL6 (involved in neutrophil chemotaxis), indicating neutrophil recruitment [78]. Histological findings confirmed macrophage aggregation and PP phagocytosis. Other in vivo studies comparing PP, PS, and PE exposures revealed polymer-specific immune responses: PS activated TLR4/NLRP3/NF-κB signaling, increasing IL-1β, IL-6, and chemokines such as MCP-1 and MIP-2, while PE upregulated TLR2 and inflammatory cells, confirming polymer-specific immune effects [90,91,92,93].

In murine models of asthma, MP exposure increased eosinophil, lymphocyte, and macrophage counts, alongside elevated IgE, IL-4, IL-5, and TNF-α levels, consistent with a Th2-type inflammatory response [26]. Gene expression analyses revealed upregulation of immune activation and stress-related genes, promoting apoptosis, altered protein folding, and impaired tissue repair. Although airway hyperreactivity remained unchanged, mucus production was higher in asthmatic mice exposed to MPs compared to controls, suggesting a role in mucus hypersecretion and airway remodeling [26]. Additional studies also reported elevated TNF-α and TGF-β expression and shorter inspiratory times following inhalation, supporting the role of MPs in inflammatory, fibrotic, and necrotic pulmonary injury [26,84].

Exposure to MPs has been associated with cellular and molecular alterations suggestive of pulmonary fibrosis. In vitro experiments on human lung epithelial cells cultured with PES spheres showed significant morphological changes and reduced proliferation, indicating inflammatory stress and cytotoxicity [21,27,59,94]. MPs were also shown to damage alveolar epithelial cells, promote fibroblast proliferation, and enhance epithelial–mesenchymal transition, leading to excessive collagen deposition and fibrotic remodeling [20,78]. Clinically, Alpaydin et al. detected higher MP concentrations in BALF from patients with interstitial lung disease, particularly in those with fibrotic phenotypes [20].

Several mechanistic pathways have been proposed to explain fibrosis induced by MP exposure. The TGF-β signaling pathway activates fibroblasts and upregulates α-SMA, vimentin and pro-fibrotic genes such as TGF-β1, CTGF and fibronectin [95,96]. The Wnt/β-catenin pathway was found to be upregulated in murine models following intratracheal exposure to MPs, promoting β-catenin nuclear translocation and transcriptional activation of fibrogenic targets [95]. The cGAS-STING pathway, activated by mitochondrial and nuclear DNA damage, triggers innate immune activation and mitochondrial dysfunction, reinforcing fibrotic injury [74,97]. Other associated mechanisms, such as Toll-like receptor/NF-κB signaling, oxidative stress responses, epigenetic remodeling, and cellular senescence, may act synergistically to sustain chronic inflammation and fibrogenesis [98,99,100,101,102,103,104,105,106,107,108,109,110].

Collectively, these findings indicate that MP exposure can initiate or amplify pro-fibrotic signaling cascades through multiple pathways, primarily involving epithelial injury, oxidative stress and dysregulation of cellular repair mechanisms (summarized in Table 3).

In 1998, Pauly et al. first demonstrated the presence of synthetic fibres in human lung tissue, particularly in cancerous samples [64]. Subsequent studies revealed that MPs can alter the composition and biophysical properties of pulmonary surfactant (LS), thereby impairing lung function [27,84,111]. Upon contacting LS, MPs increase surface tension by adsorbing phospholipids and forming protein coronas, which disrupt the structural organization of the surfactant layer [84,112]. PES particles, in particular, promote sticky aggregate formation and decrease phospholipid concentration, whereas aged MPs exert stronger effects due to the presence of oxygen-containing functional groups [84,112].

Two complementary oxidative mechanisms have been described. First, surface-mediated redox reactions on microplastic surfaces can promote the oxidation of ascorbic acid to dehydroascorbic acid, generating hydrogen peroxide (H_2_O_2_) as a by-product that can subsequently decompose into hydroxyl radicals (•OH) [112,113]. Second, environmentally persistent free radicals (EPFRs) present on aged or carbon-rich MP surfaces transfer electrons to dissolved oxygen, producing superoxide anions and subsequently H_2_O_2_ and •OH [114,115]. Recent findings by Tan et al. (2024) confirmed that PES-NPs exhibit intrinsic peroxidase-like catalytic activity, directly mediating oxidative stress through similar redox-cycling mechanisms [116].

**Figure 1:**
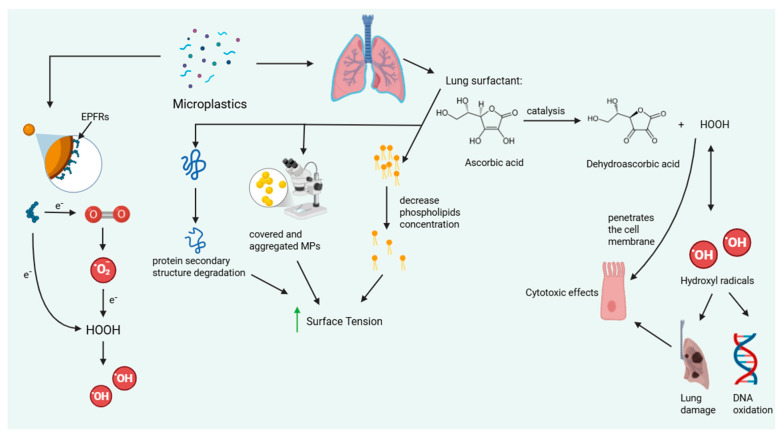
Proposed mechanism of oxidative lung injury induced by MPs.

These reactive oxygen species penetrate cell membranes and induce lipid peroxidation, protein degradation, and DNA oxidation, leading to cumulative lung injury. Collectively, these findings demonstrate that MPs increase oxidative stress and surface tension through combined chemical and physical interactions with lung surfactant (Figure 1).

Inhaled MPs interact with pulmonary surfactant, adsorbing phospholipids and proteins, which increases surface tension and destabilizes the surfactant film. On their surfaces, MPs catalyze the oxidation of ascorbic acid to dehydroascorbic acid, generating hydrogen peroxide (H_2_O_2_), while environmentally persistent free radicals (EPFRs) transfer electrons to oxygen to produce superoxide and subsequent hydroxyl radicals (•OH). These reactive oxygen species penetrate cell membranes, causing lipid peroxidation, protein denaturation, DNA oxidation, and progressive lung tissue damage.

Beyond surface-driven ROS production, microplastic toxicity arises from three mechanistically distinct components: (i) intrinsic polymer chemistry, (ii) leached small-molecule additives and (iii) adsorbed environmental pollutants. These components act through partially independent biological pathways and therefore should be mechanistically distinguished.

A growing body of evidence shows that polymer chemistry directly influences redox activity. Aromatic polymers, such as PS, display π–π interactions and electron-rich phenyl groups that facilitate redox cycling and peroxidase-like catalysis. Tan et al. showed that PS NPs exhibit intrinsic peroxidase-mimetic activity generating H_2_O_2_ and hydroxyl radicals [116]. In contrast, polymers with different electronic properties, for example, PET, with ester-linked aromatic rings, and non-aromatic polymers, such as PE, PES and PVC, exhibit distinct mechanisms of cellular interaction, more dependent on surface aging, oxidation and functional group formation than on aromatic electron transfer.

Many plastics release phthalates, bisphenols and other low-molecular-weight additives, which can exert toxicity independent of the polymer particle itself [79]. These additives can modulate inflammatory pathways or influence PPAR signaling once desorbed from the particle matrix [12]. Such effects complicate interpretation of in vitro studies, because biological responses may arise from the chemical mixture carried by the particle rather than the polymer itself.

Importantly, even chemically simple polymers can exert biological effects when sufficiently small or surface-oxidized. Wu et al. identified polyethylene fragments in human dental calculus and showed that PE NPs induce ROS generation, NF-κB activation, cytokine release, and apoptosis in gingival fibroblasts [117]. This finding reinforces that toxicity arises from the combined contribution of particle size, surface oxidation, and associated chemicals, rather than from polymer composition alone.

This mechanistic distinction helps clarify why in vivo studies, where microplastics originate from real consumer products containing additives and environmental pollutants, often reveal more complex biological responses than in vitro experiments using additive-free model particles.

Epidemiological data support these mechanistic findings. PVC exposure has long been linked to pneumoconiosis, granulomatous lung disease, fibrosis, and increased lung-cancer risk, with estimates suggesting a 20% rise in risk for each additional year of occupational exposure [79,118]. Chen et al. observed higher microfibre loads in tumoral than in adjacent normal tissue in patients with non-small-cell lung cancer, especially among older individuals or those with high environmental exposure [66]. The detected fibres, mainly polyester, rayon and cotton, measured 1–5 mm in length and were more prevalent in urban samples, reflecting environmental accumulation and prolonged persistence in lung tissue [66]. MPs may further promote carcinogenesis by sustaining chronic inflammation, interacting with macrophages, and serving as vectors for bacteria (Bacteroides, Firmicutes) and organic pollutants that modify the tumour microenvironment [66].

**Figure 2:**
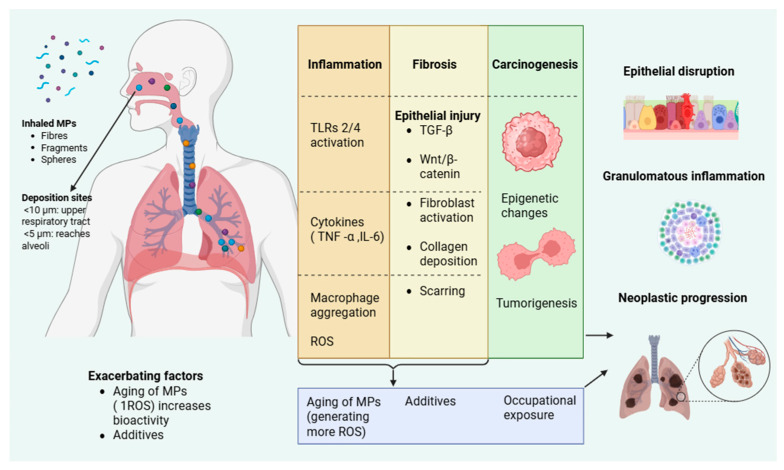
Proposed pathophysiological mechanisms of microplastic-induced respiratory injury.

Collectively, the described mechanisms demonstrate that microplastic exposure can trigger overlapping inflammatory, fibrotic, and carcinogenic pathways throughout the respiratory system. These interactions are summarized in Figure 2.

Inhaled microplastics (fibres, fragments, spheres) deposit along the respiratory tract depending on particle size, with smaller particles (<5 μm) reaching the alveoli. Surface aging and plastic additives enhance their oxidative and inflammatory potential. Microplastics activate Toll-like receptors (TLR2/4), promote cytokine release (TNF-α, IL-6), and generate reactive oxygen species (ROS), leading to macrophage aggregation and epithelial disruption. Persistent injury induces pro-fibrotic signaling (TGF-β, Wnt/β-catenin) and collagen deposition, while chronic oxidative and epigenetic changes contribute to malignant transformation.

While most studies focus on lower airway deposition, MPs also affect the nasal and upper respiratory mucosa. Nasal deposition efficiency increases during slow breathing, whereas rapid airflow promotes impaction in the upper airways [74]. MPs can irritate the nasal, pharyngeal, and laryngeal epithelium, leading to coughing and sneezing [10]. Clinical evidence indicates higher MPs counts in NLF of patients with chronic rhinosinusitis compared to healthy controls [119].

At the cellular level, both pristine PS and non-pristine PET are internalized by primary nasal epithelial cells, where they induce ROS production, mitochondrial dysfunction, and inhibition of autophagy [79]. MPs also disrupt the commensal nasal microbiota, increasing the abundance of Klebsiella and Helicobacter species and reducing Bacteroides, patterns associated with allergic rhinitis (decreased Bacteroides abundance) and chronic airway disease (Klebsiella presence) [88,120,121,122,123,124,125].

Evidence suggests that MPs may contribute to the etiology of acute rhinitis, as individuals with allergic rhinitis have been found to exhibit higher concentrations of MPs per milliliter of NLF compared with both healthy controls and patients with chronic rhinitis [73]. Host-specific factors such as MP tolerance, mucosal sensitivity and genetic predisposition likely modulate these responses. Moreover, the increased mucociliary clearance and nasal secretions characteristic of chronic rhinitis may facilitate MP elimination, resulting in lower inflammation and particle accumulation compared with acute rhinitis [73].

Paplińska-Goryca et al. investigated nasal epithelial responses in individuals with asthma and COPD exposed to PA MPs [85]. Although direct cytotoxicity was absent, MPs significantly upregulated pro-inflammatory markers: in asthmatic cells, CD24, CD193, and EGFR were overexpressed, linking PA exposure to Th2-type inflammation, whereas COPD macrophages showed increased TLR4 and IL-10 signaling, indicating oxidative stress-mediated barrier disruption. These processes collectively promote epithelial–mesenchymal transition, fibrosis, and possibly carcinogenesis [85].

Overall, upper-airway studies demonstrate that MP exposure triggers oxidative stress, inflammation and microbial alterations comparable to those seen in the lower respiratory tract. Individuals with pre-existing airway disease, such as asthma, COPD or chronic rhinitis, appear particularly vulnerable to MP-induced epithelial damage and immune dysregulation.

## 5. Conclusions

Recent evidence confirms the existence of airborne microplastics and their presence in multiple human respiratory samples, highlighting their potential clinical relevance. Both in vivo and in vitro studies suggest that inhaled MPs can penetrate deeply in the respiratory tract, interact with lung surfactant and trigger mitochondrial dysfunction, inflammation, oxidative stress, fibrogenesis and carcinogenetic processes. Moreover, MPs’ ability to absorb and carry environmental pollutants and toxic additives amplify their pathogenicity.

Despite plausible mechanistic links between MPs and respiratory diseases, current research remains heterogenous, fragmented and limited in scale. Most available studies are experimental, and epidemiological data are still lacking. Future research should prioritize standardized detection methods, long-term exposure models and a more detailed elucidation of the molecular pathways through which MPs exert toxicity.

To develop effective preventive strategies and protect public health, airborne microplastics must be formally recognized as an emerging pollutant. Strengthening environmental regulations and shaping informed public health policies will be essential for reducing human exposure and understanding the full impact of microplastics on respiratory health.

## Figures and Tables

**Table 1:** Characteristics of Airborne Microplastic Studies Conducted in Urban Environments.

Country	Analytical Method	Sample	Size	Shape	Polymer Type	Reference
United Kingdom	Fluorescence microscopy + micro-FTIR Spectroscopy	Total atmospheric deposition samples	mean 905 ± 641 μm	Fibres, fragments, films, granules, foams	Fibrous: Polyacrylonitrile, PET, PA, PP, polyurethane, cellulose Non-fibrous: PS, PP, PE, PET, PVC, polyurethane, resin	[36]
France	Visual microscopy (stereomicroscope)	Total atmospheric fallout	100–5000 µm	Mainly fibres	-	[51]
China	Visual microscopy (stereomicroscope) + FTIR + SEM	Atmospheric fallout	50–3500 µm (100 and 500 µm)	Fibers, foams, fragments, and films	PE, PP, and PS	[52]
Iran	Fluorescence microscopy + SEM-EDX	Urban surface dust	<100 up to 5 mm (mostly between 250 and 500 μm)	Fibres, sphere, hexagonal, irregular polyhedron	-	[53]
Denmark	μFTIR spectroscopy	Indoor air	11–4800 μm (mostly below 100 μm)	Fibres, fragments	PA, PES and PP	[55]
USA	Gross microscopy + fluorescent microscopy + micro-Raman spectroscopy + (µFTIRspectroscopy	Indoor and outdoor air	Indoor: 58.6 ± 55 µm Outdoor: 104.8 ± 64.9 µm	Fibers, fragments	Indoor: PVC, PE, PS, PET	[56]

**Table 2:** Overview of studies identifying microplastics in human respiratory samples.

Country	Sample Type	Analytical Method	Polymer Type	Reference
China	NFL	Polarizing microscopy, LDIR	PVC, PA, PE	[59]
China		Polarized light microscopy, LDIR	PP, PC, PA, PE, PET	[48]
China	Sputum	Polarizing microscopy, LDIR	PC, PVC, PA	[59]
Iran		μ-Raman spectroscopy	PU, PS, PE, PA, PVC	[17]
Iran		μ-Raman spectroscopy and SEM-EDS	Mostly PES	[27]
China		LDIR, FTIR microscope	PU, PES, PC	[40]
Iran	BALF	μ-Raman spectroscopy	PE, PS, PP, PET	[17]
China		LDIR, electron microscopy	PE, PET	[62]
China		LDIR	PU, PE, PET	[63]
Spain		µ-FTIR, SEM-EDS	Rayon, PES, cellulose, cotton, synthetic wool	[37]
Turkey		μ-Raman spectroscopy	PA, PET, PVC, PU, PES	[20]
Iran		μ-Raman spectroscopy and SEM-EDS	PES, PA, PET	[27]
Iran	Pleural fluid	µ-Raman, and SEM-EDS	PES, PA, PET	[27]
Brazil	Lung tissue	µ-Raman and µFTIR spectroscopy	PP, PE, cotton, PVC, cellulose Acetate, PA, PS, PU	[13]
UK		µFTIR spectroscopy	PP, PET, resin, PE	[65]
China		µFTIR, Raman spectroscopy	Cotton, rayon, PES, PET, resin	[66]

**Table 3:** Possible mechanisms for lung fibrosis caused by MPs.

Pathway	Proposed Mechanisms	Reference
TGF-β signaling	Epithelial injury increases vimentin and α-SMA, activating fibroblasts. MPs induce epithelial-to-mesenchymal transition and up-regulate pro-fibrotic genes (TGF-β1, CTGF, collagen I and fibronectin).	[95,96]
Prostaglandin E2 (PGE2)	Reduced PGE2 levels in rat lungs by styrene oxide. Antioxidants and anti-inflammatory administration increase PGE2 levels.	[97,98]
Notch	MPs activate Notch and TGF-β pathways through producing oxidative stress.	[99]
Hedgehog (SHH)	MPs activate SHH signaling pathway, promoting migration of bronchial smooth muscle cells through Gli1-mediated Snail transcription.	[100]
Midkine (MDK)	Bleomycin exposure increases MDK, collagen, α-SMA, TNF-α and TGF-β expression, but also lymphocyte percentage.	[101]
Toll-like receptors/NF-jB signaling	MPs increase TLR2 expression and activate NF-jB, resulting in inflammatory cytokine release, oxidative stress and apoptosis. Intratracheal MP exposure also elevates LPS and impairs lung function, with TLR4 upregulation and Gram-negative infection.	[102,103,104]
Epigenetic changes	MPs alter DNA-methylation profiles (e.g., zebrafish), reorganize the actin cytoskeleton (via Twf1 and F-actin), and modify expression of fibrosis-related genes, mimicking fibrotic remodeling.	[105,106]
Cellular senescence	MPs induce senescence-associated β-galactosidase activity in alveolar epithelial cells and mesenchymal stem cells (exposed to PET), leading to reduce regeneration and persistent fibrosis.	[107,108,109,110]

## Data Availability

No new data were created or analyzed in this study. Data sharing is not applicable to this article.

## Abbreviations

The following abbreviations were used in this manuscript:

As	Arsenic
ATR-FTIR	A non-destructive spectroscopic technique that uses an Attenuated Total Reflectance accessory with a Fourier Transform Infrared spectrometer
BALF	Bronchoalveolar lavage fluid
Cd	Cadmium
Cu	Cooper
Fe	Iron
μ-FTIR	micro-Fourier Transform Infrared spectrometer
LDIR	Laser Direct Infrared
LS	Lung surfactant
MDK	Midkine
MesPs	Mesoplastics
MFs	Microfibres
MMAD	Mass median aerodynamic diameter
Mn	Manganese
MPs	Microplastics
NH4+	Amino group
Ni	Nickel
NLF	Nasal lavage fluid
NPs	Nanoplastics
•OH	Hydroxyl radicals
PA	Polyamide
PC	Polycarbonate
PE	Polyethylene
PES	Polyester
PET	Polyethylene terephthalate
PGE2	Prostaglandin E2
PO3-	Phosphoric acid
PP	Polypropylene
PPARs	Peroxisome-proliferator-activated receptors
PS	Polystyrene
PU	Polyurethane
PVC	Polyvinyl chloride
ROS	Reactive oxygen species
SEM-EDS	Scanning electron microscopy with energy-dispersive X-ray spectroscopy
SHH	Hedgehog
UNEP	United Nations Environment Programme
Zn	Zinc
